# AdipoR1 Regulates Ionizing Radiation-Induced Ferroptosis in HCC cells through Nrf2/xCT Pathway

**DOI:** 10.1155/2022/8091464

**Published:** 2022-06-13

**Authors:** Hao Feng, Yi Liu, Yuhan Gan, Mengke Li, Rui Liu, Zhenzhen Liang, Lianchang Liu, Lan Li, Huajian Chen, Guanghui Li, Zhujun Tian, Xiaodong Liu, Shumei Ma

**Affiliations:** ^1^School of Public Health and Management, Wenzhou Medical University, Wenzhou, Zhejiang 325035, China; ^2^NHC Key Laboratory of Radiobiology (Jilin University), Changchun, Jilin 130021, China; ^3^The second hospital of Ji Lin University, Changchun, Jilin 130021, China; ^4^Key Laboratory of Watershed Science and Health of Zhejiang Province, Wenzhou Medical University, Wenzhou, China; ^5^South Zhejiang Institute of Radiation Medicine and Nuclear Technology, Wenzhou, Zhejiang 325035, China

## Abstract

Radiotherapy has been used for decades in the treatment of liver cancer. We previously found that adiponectin receptor (AdipoR1) is a prognostic biomarker for hepatoma carcinoma (HCC) after stereotactic body radiation therapy (SBRT) and blocking AdipoR1 enhances radiation sensitivity in hepatoma carcinoma cells. In the current study, we aimed to elucidate the roles of AdipoR1 in ionizing radiation- (IR-) induced radiosensitivity by activating ferroptosis pathway in HCC cells. We found that IR upregulated the expression of AdipoR1 and furthermore promoted the protein stability of transcription factor Nrf2, Nrf2 binded to the xCT promoter and increased xCT transcription and expression, and this directly contributed to the protective function in the early stage of radiation in HCC cells. AdipoR1 knockdown significantly inhibited expression of Nrf2 and xCT and, furthermore, increased both IR- and erastin-induced ferroptosis, which could be abolished by the rescue of Nrf2 and xCT. For the first time, we found that radiation-induced ferroptosis was mediated by AdipoR1-Nrf2-xCT pathway in HCC cells. These results provide new insights to the development and application of novel therapeutic strategies for hepatoma carcinoma.

## 1. Introduction

Hepatocellular carcinoma (HCC) is the most common primary liver cancer with poor prognosis, and it is also the third cause of global cancer-related death [1–4]. Most patients with HCC are usually diagnosed at late stage and usually receive local radiotherapy and chemotherapy [5, 6]. However, radiotherapy still has some inherent limitations, such as side effects and radiation resistance. It is an urgent task to explore novel targets that improve the radiosensitivity of HCC.

Ionizing radiation (IR) induces DNA double-strand breaks to trigger various modes of cell death, including apoptosis, necrosis, autophagy, ferroptosis, and mitotic catastrophe [7]. Ferroptosis is an iron-dependent form of regulated cell death induced by excessive lipid peroxidation [8–10]. Recent studies have shown that ferroptosis plays an important role in tumor suppression and cancer treatment resistance [11–15].

Ferroptosis is regulated by a network revolving around glutathione peroxidase 4 (GPX4). Correspondingly, genetically or pharmacologically inactivating GPX4 or xCT can lead to ferroptosis [8, 16, 17]. The function of cystine/glutamate antiporter xCT is to import cystine for glutathione biosynthesis and antioxidant defense, and it is overexpressed in multiple human cancers. Thus, the mechanism of ferroptosis agonists (such as erastin) is to inhibit systemic xc−-mediated cystine import, resulting in depletion of intracellular GSH and subsequent iron-dependent lipid peroxidation [18, 19]. Nuclear factor E2-related factor 2 (Nrf2) is a key regulator required for cells to maintain oxidative homeostasis, which is activated under conditions of high oxidative stress. Nrf2 protects cells from oxidative stress by binding to antioxidant response elements (AREs) in the nucleus, thereby promoting transcription of target genes and translation of antioxidant and anti-inflammatory proteins [20]. In addition, studies have shown that Nrf2 can inhibit ferroptosis by upregulating the expression of xCT, and knocking down Nrf2 can significantly reduce the level of xCT and promote the accumulation of lipid peroxides [21].

Adiponectin is a protein with insulin-sensitizing, anti-inflammatory, and antiapoptotic functions [22]. Adiponectin needs to bind to its receptor to exert its biological function. At present, 3 kinds of adiponectin receptors were proven: AdipoR1, AdipoR2, and T-cadherin. Studies have shown that adiponectin receptors are closely related to the occurrence and development of various cancers, such as breast cancer, colorectal cancer, and renal cell carcinoma [23, 24].

In our previous work, we followed up and collected blood samples from liver cancer patients with SBRT treatment. After transcriptome analysis, we found that AdipoR1 was significant increased and might be a prognostic biomarker for HCC after SBRT. However, how AdipoR1 regulates the progression of HCC and its underlying mechanisms remains unknown. In this study, we found that AdipoR1 is highly expressed in tumor cells, and its knockdown could enhance radiosensitivity of HCC cells via Nrf2-xCT signaling pathway. These findings suggested that AdipoR1-regulated ferroptosis may be an important target for enhancing the radiosensitivity of HCC, which also provides a new strategy for reducing the radioresistance of HCC in the future.

## 2. Materials and Methods

### 2.1. Reagents and Antibodies

Trypan blue solution (Prod. No. T8154), erastin (Selleck Chemicals, USA), ferrostatin-1 (Fer-1), ZVAD-FMK, 3-methyladenine (3-MA), and rapamycin (Rapa) were purchased from MedChemExpress (MCE, USA), and phosphatase inhibitor cocktails 2 and 3 (Prod. No. P5726 & P0044) were from Sigma-Aldrich (St. Louis, MO, USA). A protease inhibitor cocktail (ref no. 11836 153 001) was from Roche Diagnostics (Basel, Switzerland). Primary antibodies are as follows: AdipoR1 (ab126611), transferrin (ab8241), and GPX4 (125066) from Abcam (Cambridge, MA, USA); xCT (#12691S), CD71 (#13113S), and Nrf2 (#12721S) from Cell Signaling Technology (Danvers, MA USA); GAPDH from Proteintech (Rosemont, IL); actin (A3853) from Sigma; and AdipoR1 (SC-518030) from SANTA. Secondary antibodies are as follows: goat anti-rabbit IgG (H+L)-HRP conjugate (Cat. No. 170-6515) and goat anti-mouse IgG (H+L)-HRP conjugate (Cat. No. 170-6516) obtained from Bio-Rad Laboratories (Mississauga, ON, Canada). siRNA, targeting human xCT, and the corresponding control, siRNA NC, were purchased from GenePharma (Shanghai, China).

### 2.2. Cell Culture

Human hepatocellular carcinoma cell lines MHCC-97H and HepG2 and normal hepatic cells LO2 were purchased from the Cell Bank of the Chinese Academy of Sciences (Beijing, China) and confirmed by short tandem repeat (STR). The cell was cultured in Dulbecco's modified Eagle's medium mixture medium (Invitrogen Inc.) supplemented with 10% fetal bovine serum (Invitrogen Inc.) and 1% penicillin/streptomycin (cat. no. 10378016, Life Technologies) in a humidified 5% CO_2_, 37°C incubator.

### 2.3. Radiation

The cells were exposed to ionizing radiation (10 Gy) using an X-ray generator (X-RAD 320 ix, Precision X-ray Inc., North Branford, CT, USA) at a dose rate of 3 Gy/min.

### 2.4. Cell Viability by CCK-8 Assays

Cell viability was determined by Cell Counting Kit-8 (CCK-8, Dojindo Laboratories, Japan) according to the manufacturer's protocol. The cells were seeded in 96-well plates (2 × 10^3^ cells/well) and treated with drugs. CCK-8 was added to each well, and the cells were incubated for 3 h. OD values were recorded at 450 nm using a microplate reader. The proliferation rate of the cells was calculated by the following formula: cell viability = (OD experimental group − OD blank/OD control group − OD blank) × 100%.

### 2.5. Flow Cytometric Analysis of Cell Death

Trypan blue solution was used for observation of cell death by flow cytometry. The cells were seeded in 6-well plates (8 × 10^4^ cells/well) and irradiated with 10 Gy. The cells were collected and centrifuged at 500 × *g* for 5 minutes at 4°C. The cell pellet was washed with PBS, stained with trypan blue for 3 minutes, and detected cell death by flow cytometry (ACEA NovoCyte 2040R, USA).

### 2.6. Colony Formation Assay

The cells were placed in 6-well plates and cultured in DMEM (Invitrogen) containing 10% FBS (Gibco) at 37°C and 5% CO_2_. 24 h later, the cells were irradiated with different dose, as 0, 2, 4, and 6 Gy. After incubation for 14 days, the colonies were fixed with 4% paraformaldehyde (Solarbio, Beijing, China) for 30 min and stained with 0.2% crystal violet (Solarbio, Beijing, China) at room temperature for 15 min. The colonies containing ≥50 cells per dish were counted. Cell survival curves were fitted with the multitarget, single-hit model.

### 2.7. Lipid Peroxidation Detection

The cells (2 × 10^5^) in 6-well plates were incubated with 1 ml of fresh medium containing 5 *μ*M of BODIPY 581/591C11 (Invitrogen) at 37°C in the dark for 30 min. The cells were then trypsinized, washed, and resuspended in 0.2 ml of PBS for flow cytometry analysis. A minimum of 20,000 cells were analyzed per condition.

### 2.8. siRNAs Infection and Establishment of Cell Lines

Lentiviral short hairpin RNA (shRNA) vector targeting AdipoR1 (pLKO.1-shAdipoR1) was constructed according to the protocol of pLKO.1-blasticidin vector (Addgene, Cambridge, MA, USA). Briefly, the forward oligo CCGGCGTCTATTGTCATTCAGAGAACTCGAGTTCTCTGAATGACAATAGACGTTTTTG and reverse oligo AATTCAAAAACGTCTATTGTCATTCAGAGAACTCGAGTTCTCTGAATGACAATAGACG were annealed and inserted into the pLKO.1-blasticidin vector. Lentiviruses were produced in 293T cells after cotransfection of pLKO.1-shAdipoR1 or pLKO.1-shScramble, packing plasmid psPAX2, and envelope plasmid pMD2G. The supernatant containing viruses was collected 48 h after transfection, filtered, and used for infecting target cells in the presence of 10 *μ*g/ml of polybrene (Sigma-Aldrich, H9268) prior to drug selection with 7 *μ*g/ml of blasticidin for one week.

### 2.9. Construction of Expression Vectors

The xCT and Nrf2 cDNA were PCR amplified and cloned into pcDNA3.1Flag at BamHI and XhoI sites, respectively.

### 2.10. Luciferase Reporter Assays

Reporter plasmids of xCT promoter double luciferase was synthesized from Generalbiol (Anhui, China) and inserted into the pGL3-Basic vector. pGL3-Basic xCT promoter and pGL3-Basic vector were cotransfected into cells with PCDNA3.1-Flag Nrf2, respectively. 48 hours after transfection, luciferase activity was determined on a Centro LB 960 Luminometer (Berthold Technologies, Germany), and the activity of renilla luciferase was used as a standardized control.

### 2.11. qRT-PCR

Total RNA was extracted from cells with TRIzol solution (TaKaRa, Dalian, China). PrimeScriptTM RTMaster Mix (TaKaRa, Dalian, China) was used for reverse transcription. qRT-PCR was carried out on a QuantStudio real-time PCR instrument (Thermo Fisher Scientific, USA) using SYBR Premix Ex Taq II (TaKaRa). The conditions of thermal cycling were as follows: 95°C for 30 s followed by 40 cycles at 95°C for 5 s and at 60°C for 30 s. The primers are shown in Table 1, and the 2^−*ΔΔ*Ct^ was utilized to calculate the relative expression levels.

### 2.12. Western Blot Analysis

The cells were collected with a cell scraper and lyzed with RIPA buffer. Total proteins (20 *μ*g) were separated on a 12% SDS-PAGE and transferred onto PVDF membrane (Millipore). After blocking with 6% skim milk in Tris-buffered saline-tween (TBST) for 1 h at room temperature, the membranes were incubated overnight at 4°C with primary antibodies, washing 3 times followed by incubation with secondary antibody for 1 h at room temperature. The immunoreactions were visualized by ECL solution (Thermo Fisher Scientific, USA) and analyzed using the ImageJ software (Bio-Rad).

### 2.13. Statistical Analysis

SPSS 22.0 software (SPSS, Chicago, IL, USA) was utilized for statistical analyses. Differences between the two groups were evaluated with Student's *t*-test (two-tailed). One-way ANOVA followed by Bonferroni post hoc tests was performed to analyze multiple groups. Survival curve was generated with Kaplan–Meier method. Experimental results are presented as mean ± SD. Data was considered as statistically significant when *p* value was less than 0.05.

## 3. Result

### 3.1. IR Upregulated the Expression of AdipoR1 in HCC Cells

**Figure 1:**
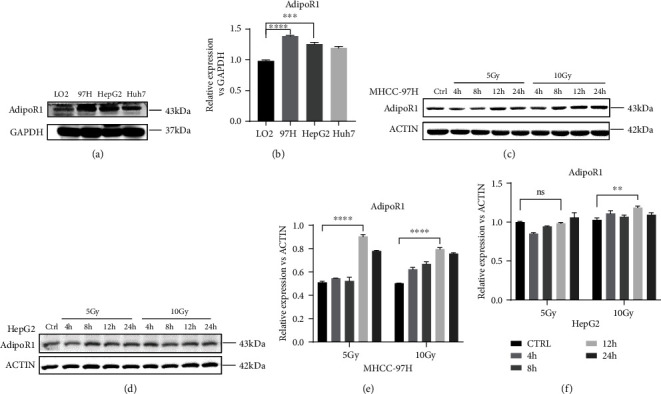
IR upregulated the expression of AdipoR1 in HCC cells. (a and b) Western blotting showed the basal expression level of AdipoR1 in HCC cells and normal hepatic cells LO2. (c–f) Western blotting showed the expression level of AdipoR1 after IR (5 Gy and 10 Gy) in MHCC-97H and HepG2 cells. Data is presented as mean ± SD. ^∗^*p* < 0.05, ^∗∗^*p* < 0.01, and ^∗∗∗^*p* < 0.001.

Referring to the TCGA, AdipoR1 mRNA level was higher in tumor as compared with normal tissues (Fig. S1A). K-M curve showed that high expression of AdipoR1 was related to poor prognosis in HCC patients (Fig. S1B). Furthermore, the basal expression of AdipoR1 in HCC cells was tested and remarkably higher than hepatic cells (Figures 1(a) and 1(b)). Next, we analyzed the expression of AdipoR1 under IR in HCC cells; 10 Gy X-rays increased the expression of AdipoR1 in both MHCC-97H and HepG2 cells compared with sham (Figures 1(c)–1(f)). Taken together, these results demonstrated that radiation could upregulate AdipoR1 expression in HCC cells.

### 3.2. AdipoR1 Knockdown Increased Radiosensitivity of HCC Cells

**Figure 2:**
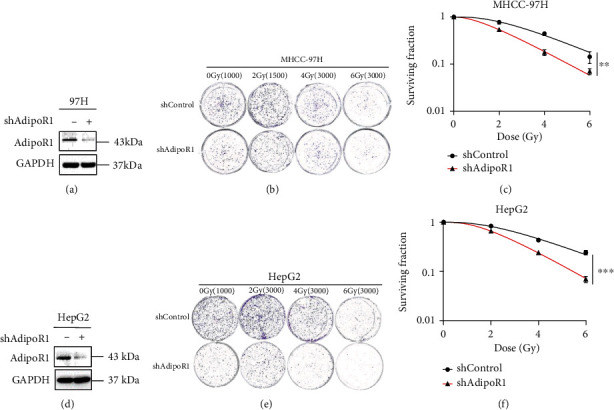
AdipoR1 knockdown increased radiosensitivity of HCC cells. (a and d) AdipoR1 knockdown was determined by Western blot in MHCC-97H (a) and HepG2 (d) cells. (b–f) After knockdown of AdipoR1, sensitization of radiation-treated MHCC-97H (b and c) and HepG2 (e and f) cells was evaluated through colony formation assay, respectively. The sensitization of radiation was measured using the multitarget, single-hit model. Data is presented as mean ± SD. ^∗^*p* < 0.05, ^∗∗^*p* < 0.01, and ^∗∗∗^*p* < 0.001.

Radiotherapy uses high-energy ionizing radiation to produce DNA double-strand breaks that induce various modes of cell death, including apoptosis, necrosis, autophagy, and mitotic catastrophe. To investigate the effects of AdipoR1 on radiation-induced cell death, we generated AdipoR1 knockdown MHCC-97H and HepG2 cells by shRNA. The effect of AdipoR1 knockdown was validated by Western blot (Figures 2(a) and 2(d)). AdipoR1 knockdown increased radiation-induced cell death in both of MHCC-97H (Fig. S2B-C) and HepG2 (Fig. S2E-F) cells.

To further determine the effects of AdipoR1 on radiosensitivity, colony formation assay was performed in MHCC-97H (Figures 2(b) and 2(c)) and HepG2 cells (Figures 2(e) and 2(f)). Colony formation assay revealed that AdipoR1 knockdown significantly reduced the survival rate in HCC cells, compared with shControl group. Taken together, these results demonstrated that AdipoR1 knockdown increased radiosensitivity in HCC cells.

### 3.3. AdipoR1 Is Involved in IR-Induced Ferroptosis in HCC Cells

**Figure 3:**
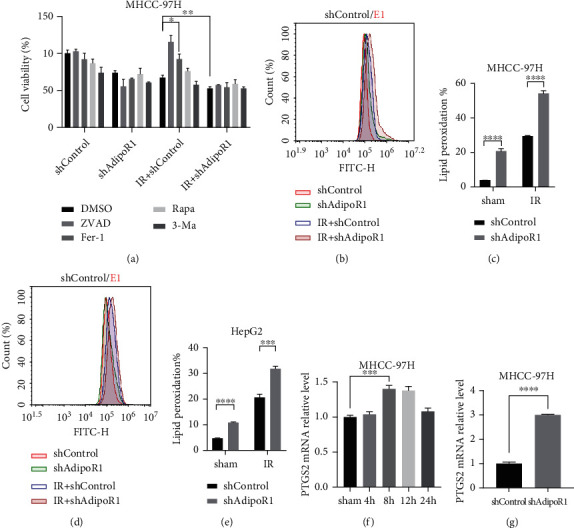
AdipoR1 is involved in IR-induced ferroptosis in HCC cells. (a) MHCC-97H cells were pretreated with different inhibitors of cell death 3-MA, ZVAD, Rapa, and Fer-1 and irradiated. The cell viability was detected by CCK-8 kit. (b–e) After treatment with 10 Gy for 48 h, lipid peroxidation was assessed by flow cytometry using C11-BODIPY in AdipoR1 knockdown MHCC-97H (b and c) and HepG2 (d and e) cells, respectively. (f and g) qRT-PCR showed the mRNA level of PTGS2 in HCC cells after radiation or AdipoR1 knockdown. Data is presented as mean ± SD. ^∗^*p* < 0.05, ^∗∗^*p* < 0.01, ^∗∗∗^*p* < 0.001, and ^∗∗∗∗^*p* < 0.0001.

It has been reported that IR induces ferroptosis in cancer cells [8]. We further investigated whether AdipoR1 regulates IR-induced ferroptosis in HCC. The cell viability of MHCC-97H with shAdiopR1 was lower than shControl following IR treatment. To investigate the types of cell death AdipoR1 involved, MHCC-97H cells with shAdipoR1 or shControl were pretreated by such inhibitors of cell death before irradiation as 3-MA, ZVAD, Rapan, and Fer-1. Compared to DMSO group, the IR-induced inhibition of cell viability was significantly reversed by Z-VAD and Fer-1 in AdipoR1 positive cells (shControl group) but failed to change in shAdipoR1 cells, indicating IR-induced apoptosis and ferroptosis in MHCC-97H cells in an AdipoR1 dependent manner (Figure 3(a)). Moreover, the hallmarks of ferroptosis include the accumulation of lipid peroxidation and ferroptosis-relative genes such as PTGS2 [8]. Simultaneously, the increase of lipid peroxidation and PTGS2 expression was also induced in shAdipoR1 cells compared with shControl cells after IR treatment (Figures 3(b)–3(f)). AdipoR1 silencing also increased the expression of PTGS2 (Figure 3(g)). These data suggested that ferroptosis occupied a very important position in IR-induced cell death and AdipoR1 acted as a key regulator of this effect.

### 3.4. AdipoR1 Is Involved in Erastin-Induced Ferroptosis in HCC Cells

**Figure 4:**
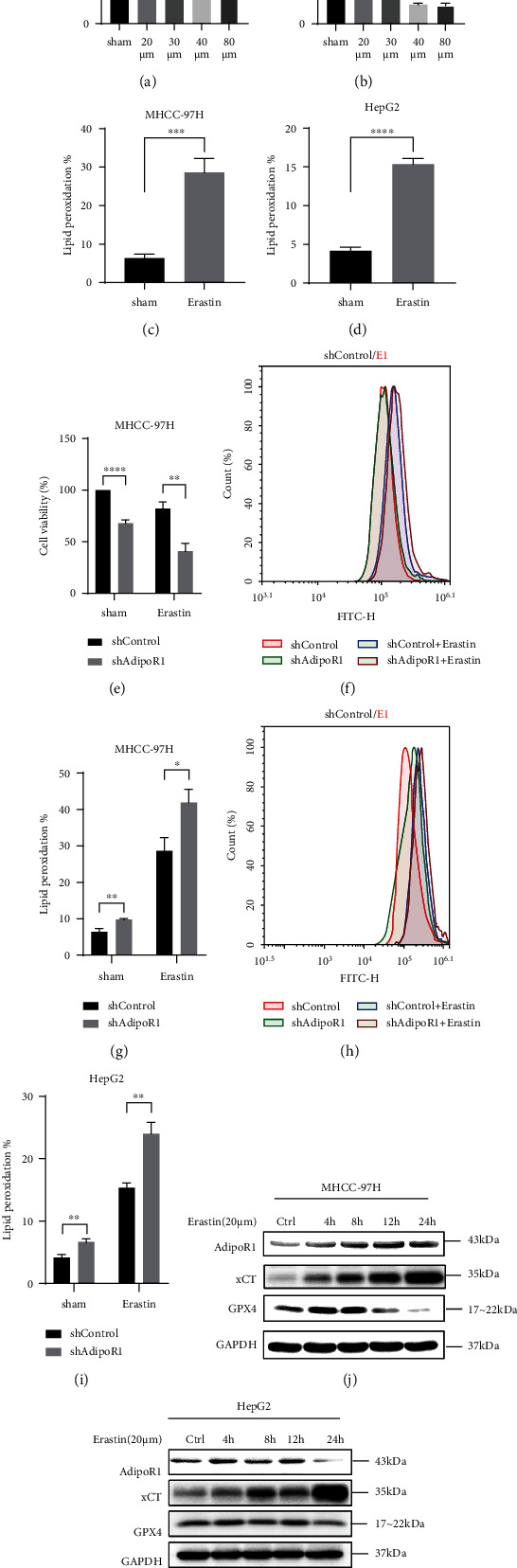
AdipoR1 is involved in erastin-induced ferroptosis in HCC cells. (a–d) Lipid peroxidation and cell viability of erastin-treated MHCC-97H and HepG2 cells were assessed by flow cytometry using C11-BODIPY (a and b, ∗ vs Ctrl) and CCK-8 kit, respectively. (e–i) After treatment with erastin (20 *μ*M) for 48 h, lipid peroxidation and cell viability were assessed in AdipoR1 knockdown MHCC-97H and HepG2 cells, respectively. (j and k) After treatment with erastin (20 *μ*M) for various times (4 h, 8 h, 12 h, and 24 h), Western blot showed the protein expression of AdipoR1, xCT, and GPX4 in HCC cells. Data is presented as mean ± SD. ^∗^*p* < 0.05, ^∗∗^*p* < 0.01, ^∗∗∗^*p* < 0.001, and ^∗∗∗∗^*p* < 0.0001.

Erastin is a classic ferroptosis activator and triggers ferroptosis in a variety of cells. To determine whether erastin could induce ferroptosis in HCC cells, we first analyzed the median lethal concentration LC50 of erastin in MHCC-97H and HepG2 cells (Figures 4(a) and 4(b)); the LC50 values of erastin are 40 *μ*M and 20 *μ*M in MHCC-97H and HepG2 cells, respectively. Next, the lipid peroxidation was detected and evaluated in MHCC-97H and HepG2 cells 48 h after erastin treatment (Figures 4(c) and 4(d)); the data showed the erastin-induced ferroptosis in HCC cells. Furthermore, to investigate whether AdipoR1 is involved in this process, AdipoR1 silencing model was used. As shown in Figures 4(e)–4(i), AdipoR1 silencing further reduced cell survival and elevated lipid peroxidation after erastin treatment (Figures 4(e) and 4(i)).

Erastin-induced ferroptosis might also involve other signals. To test this hypothesis, we examined the expression of several key ferroptosis-relative proteins in response to erastin. As shown in Figures 4(j) and 4(k), after erastin treatment, the expression of AdipoR1 and xCT was induced, while the GPX4 expression decreased in MHCC-97H and HepG2 cells. Our results verified that AdipoR1 was involved in erastin-induced ferroptosis in HCC cells.

### 3.5. Effect of AdipoR1 on Ferroptosis-Related Proteins after IR

**Figure 5:**
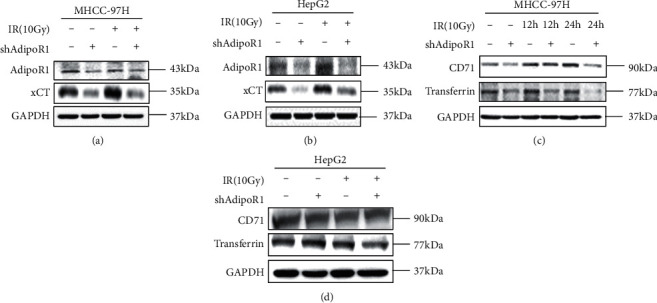
Effect of AdipoR1 on ferroptosis-related proteins in HCC cells after IR. (a–d) Western blot showed the effects of AdipoR1 on the ferroptosis-related proteins in shAdipoR1 MHCC-97H and HepG2 cells after radiation. (a and b) MHCC-97H. (c and d) HepG2.

As previously pointed out, AdipoR1 knockdown could increase IR-induced ferroptosis in HCC cells. To study the potential mechanisms, we examined the expression levels of several key protein involved in ferroptosis after IR. Transferrin is the main iron-containing protein in plasma, responsible for carrying the iron absorbed by the digestive tract and the iron released by the degradation of red blood cells. Transferrin receptor 1 (CD71) is a type II transmembrane receptor and carrier protein responsible for the uptake of cellular iron through receptor-mediated endocytosis [25]. As shown in Figures 5(a) and 5(b), AdipoR1 knockdown significantly inhibited expression of xCT, CD71, and transferrin in MHCC-97H with or without IR. AdipoR1 knockdown decreased the expression of xCT but failed to change the expression of CD71 and transferrin in HepG2 cells with or without IR (Figures 5(c) and 5(d)), suggesting that AdipoR1 regulated the expression of xCT after IR in HCC cells.

### 3.6. AdipoR1 Protected Cells from IR-Induced Ferroptosis by Upregulating xCT Expression

**Figure 6:**
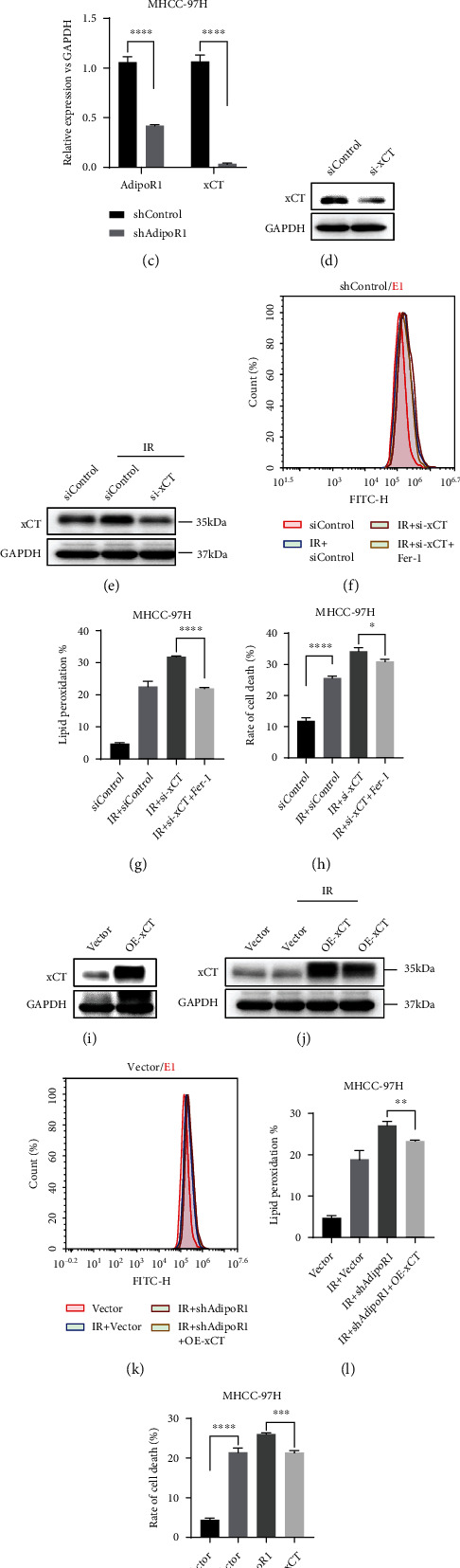
AdipoR1 protected cells from IR-induced ferroptosis by upregulating xCT. (a) TCGA data shows that the expression level of AdipoR1 is positively correlated with xCT. (b and c) Western blot showed the levels of AdipoR1 and xCT in MHCC-97H cells after knockdown of AdipoR1 by shRNA. (d–h) xCT knockdown rendered MHCC-97H cells susceptible for ferroptosis and lipid peroxidation accumulation induce by IR. (i–m) Overexpression of xCT rescue IR-induced ferroptosis in shAdipoR1 cells. Data is presented as mean ± SD. ^∗^*p* < 0.05, ^∗∗^*p* < 0.01, ^∗∗∗^*p* < 0.001, and ^∗∗∗∗^*p* < 0.0001.

To investigate the relationship between the protein expression of AdipoR1 and xCT, we analyzed publicly available gene expression datasets and found that the AdipoR1 expression level positively correlated with the xCT level in two cohorts of hepatoma carcinoma patients (Figure 6(a)). Compared with the cells in shControl group, the xCT protein levels were dramatically decreased in the shAdipoR1group (Figures 6(b) and 6(c)). This correlation is of significance given that xCT overexpression found in lung adenocarcinoma [26], colorectal cancer [27], and HCC cells [28] has been shown to promote the growth of the tumors. To further verify whether AdipoR1 regulates ferroptosis via xCT after IR, xCT knockdown increased IR-induced lipid peroxidation and cell death, which could be significantly rescued by Fer-1 (Figures 6(d)–6(h)). Subsequently, xCT was overexpressed through transfection with pcDNA3.1Flag-xCT; overexpression of xCT successfully suppressed IR-induced lipid peroxidation and cell death in HCC cells (Figures 6(i)–6(m)). These results suggested that AdipoR1 protected cells from IR-induced ferroptosis by promoting the expression of xCT, which may be its key downstream target.

### 3.7. AdipoR1 Regulated IR-Induced Ferroptosis by AdipoR1-Nrf2-xCT Pathway

**Figure 7:**
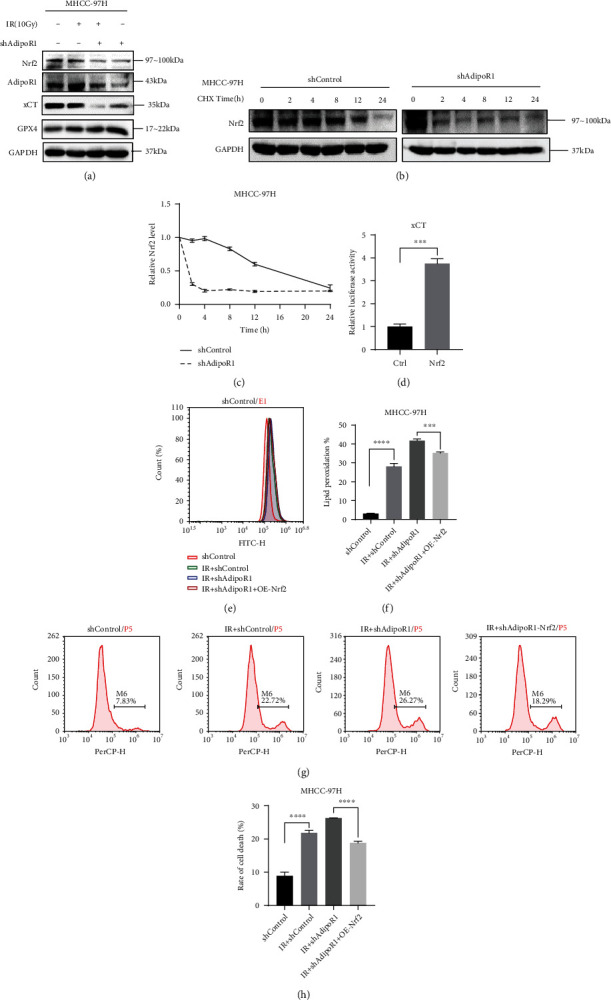
AdipoR1 regulated IR-induced ferroptosis by AdipoR1-Nrf2-xCT pathway. (a) The expression of Nrf2 and GPX4 in shControl and shAdipoR1 MHCC-97H after 10 Gy radiation. (b and c) The expression of Nrf2 was tested in shControl and shAdipoR1 MHCC-97H treated with CHX (100 *μ*g/ml). (d) Effect of Nrf2 on xCT transcription activity in 293T cells. (e–h) Lipid peroxidation and cell death were assessed in shControl and shAdipoR1with or without Nrf2 overexpression. Data is presented as mean ± SD. ^∗^*p* <0.05, ^∗∗^*p* < 0.01, ^∗∗∗^*p* < 0.001, and ^∗∗∗∗^*p* < 0.0001.

Previously, Nrf2 exert its antioxidant role in cellular protection by regulating xCT expression [21]. In addition, we further explored the molecular mechanism of AdipoR1 in regulating xCT. Firstly, we determined whether AdipoR1 knockdown influenced the expression of Nrf2. As shown in Figure 7(a), the expression of Nrf2 and xCT decreased in shAdipoR1 MHCC-97H. To determine whether AdipoR1 regulates the Nrf2 stability, the cells were treated with protein synthesis inhibitor cycloheximide (CHX) for different periods of time. CHX treatments significantly reduced Nrf2 protein levels in shAdipoR1 MHCC-97H cells (Figures 7(b) and 7(c)), indicating that AdipoR1 knockdown decreased NRF2 protein stability. Moreover, luciferase reporter assay determined that Nrf2 could bind to xCT promoter and improve the transcription of xCT (Figure 7(d)). Subsequently, overexpression of Nrf2 successfully reduced the IR-induced lipid peroxidation and cell death in shAdipoR1 cells (Figures 7(e)–7(h)). These results suggested that AdipoR1 protected cells from IR-induced ferroptosis by promoted the expression of Nrf2 and, furthermore, increased the transcription and expression of xCT. Therefore, AdipoR1 might regulate IR-induced ferroptosis by AdipoR1-Nrf2-xCT pathway in HCC cells.

## 4. Discussion

Radiotherapy has been used for decades in the treatment of liver cancer. For early stage liver cancer, radiotherapy is mainly used for patients who cannot be surgically removed or ablated; for intermediate and advanced liver cancer, radiotherapy is mainly combined with other therapies. However, the problem of radioresistance in current radiotherapy leads to poor prognosis.

AdipoR1 is an adiponectin receptor, which participates in regulation of glucose and lipid metabolism through stimulation of fatty acid oxidation, suppression of hepatic glucose output, and increased insulin sensitivity in liver [29]. In our previous work, we found that AdipoR1 is a prognostic biomarker for HCC after SBRT in primary liver cancer and blocking AdipoR1 enhances radiation sensitivity in hepatoma carcinoma cells both in vitro and in vivo [30]. In this study, we aimed to understand the mechanisms regulating the radioresistance and figured out what type of cell death did AdipoR1 participate in. We found that knockdown of AdipoR1 increased IR-induced increased cell death and that Fer-1, a ferroptosis inhibitor, significantly reduced cell death. We then examined ferroptosis indicators lipid ROS and PTGS2, both of which we found were also significantly increased in the AdipoR1 knockdown group after IR. These results were also consistent with what we detected after treatment of HCC cells with erastin (an inducer of ferroptosis). Taken together, AdipoR1 is involved in ionizing radiation-induced ferroptosis in HCC cells.

Ferroptosis is a nonapoptotic form of cell death that can be induced by metabolic stress such as GSH depletion [8]. Many studies have shown that ferroptosis can selectively target aggressive cancer stem cells and is also expected to enhance the efficacy of radiotherapy and overcome the resistance [11, 12, 31–34]. However, the mechanism by which AdipoR1 regulated IR-induced ferroptosis remains unclear. Recent studies revealed that xCT overexpression inhibits ferroptosis through importing cystine, promoting GSH biosynthesis, and subsequently facilitating GPX4-mediated detoxification of lipid peroxides [9]. GPX4, a glutathione peroxidase, utilizes reduced glutathione to convert lipid hydroperoxides to lipid alcohols, thereby mitigating lipid peroxidation and inhibiting ferroptosis [16, 17, 35]. We then examined the expression levels of several key proteins involved in ferroptosis. The datas showed that AdipoR1 knockdown significantly inhibited expression of xCT in HCC cells. We also examined the expression of ferroptosis-related protein CD71 and transferrin. The results showed that the expression of CD71 and transferrin was reduced in shAdipoR1 MHCC-97H cells but had no significant effect on the expression of both proteins in HepG2 cells. This indicated that AdipoR1 mainly regulated IR-induced ferroptosis in HCC cells by regulating the expression of xCT. And overexpression of xCT could decrease the lipid ROS and cell death caused by AdipoR1 knockdown. Therefore, we suspected that AdipoR1 might regulate ferroptosis through xCT in HCC cells.

Recent studies have shown that AdipoR1 could maintain the integrity of the blood-brain barrier through the APPL1/AMPK/Nrf2 signaling pathway [36]. And Nrf2 exerts its antioxidant role in cellular protection by regulating xCT expression [21]. Therefore, we speculated that AdipoR1 could regulate the expression of xCT via Nrf2. The results showed that overexpression of Nrf2 could decrease the lipid ROS and cell death caused by AdipoR1 knockdown. And AdipoR1 could mediate the expression of Nrf2 by regulating the protein stability of Nrf2. Meanwhile, Nrf2 could regulate transcription activity of xCT. This result was consistent with the previous reports [21].

**Figure 8:**
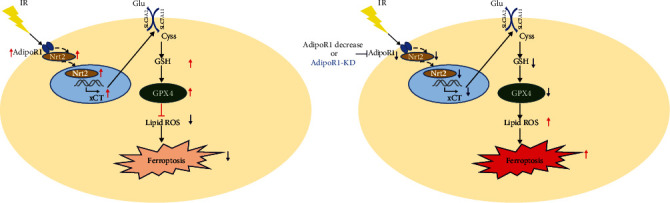
A schematic model demonstrating the roles of AdipoR1 in IR-induced ferroptosis.

In summary, our results demonstrated that AdipoR1 knockdown could enhance radiosensitivity of HCC cells via Nrf2-xCT signaling pathway (Figure 8). These findings suggest that AdipoR1-regulated ferroptosis may be an important target for enhancing the radiosensitivity of HCC, which also provides a new strategy for reducing the radioresistance of HCC in the future.

## 5. Conclusion

Together, these results suggested that AdipoR1 participated in IR/Erastin-induced ferroptosis in HCC cells through AdipoR1-Nrf2-xCT pathway.

## Figures and Tables

**Table 1:** Primers used in this study.

Gene		Forward	Reverse
Human	SLC7A11/xCT	5′-CCATGGGTGGAATCATATTGGA-3′	5′-TCAACGGATTTGGTCGTATTGG-3′
	AdipoR1	5′-CTCATCTACCTCTCCATCGT-3′	5′-GAACACTCCTGCTCTTGTCT-3′
	PTGS2	5′-TAGGATTCAGGGCTTTCACTGGCT-3′	5′-TGTCAGCCGACAATGAGATGTGGA-3′
	GAPDH	5′-GCGTGGGCATGTCTCTGAC-3′	5′-GCTGGTAATGGACCAAAGACTTC-3′

## Data Availability

All data generated or analyzed during this study are included in this published article.

## Abbreviations

GPX4: Glutathione peroxidase 4
GSH: Glutathione
ARE: Antioxidant response element
HCC: Hepatocellular carcinoma
IR: Ionizing radiation
AdipoR1: Adiponectin receptor one
AdipoR2: Adiponectin receptor two
STR: Short tandem repeat
OD: Optical density
SLC7A11/xCT: Solute carrier family 7 member 11
CD71: Transferrin receptor 1
TBST: Tris-buffered saline-tween
K-M: Kaplan–Meier
TCGA: The Cancer Genome Atlas
SBRT: Stereotactic body radiotherapy
3-MA: 3-Methyladenine
ZVAD: Z-VAD-FMK
RAPA: Rapamycin
Fer-1: Ferrostatin-1
qRT-PCR: Real-time quantitative reverse transcription PCR
ROS: Reactive oxygen species
PUFA-PLs: Polyunsaturated fatty acid-containing phospholipids.

## Supplementary Materials

Supplementary Materials Supplementary file 1: Fig. S1: high expression levels of AdipoR1 was related to poor prognosis in HCC patients. Supplementary file 2: Fig. S2: AdipoR1 knockdown increased IR-induced cell death in HCC cells.
