# Supplementary material for: AdipoR1 Regulates Ionizing Radiation-Induced Ferroptosis in HCC cells through Nrf2/xCT Pathway
Source: Oxid Med Cell Longev. 2022 Jun 13;2022:8091464. doi: 10.1155/2022/8091464 (PMC9208988; doi:10.1155/2022/8091464)
Supplement: Supplementary Materials — Supplementary file 1: Fig. S1: high expression levels of AdipoR1 was related to poor prognosis in HCC patients. Supplementary file 2: Fig. S2: AdipoR1 knockdown increased IR-induced cell death in HCC cells. [file 8091464.f1.docx]

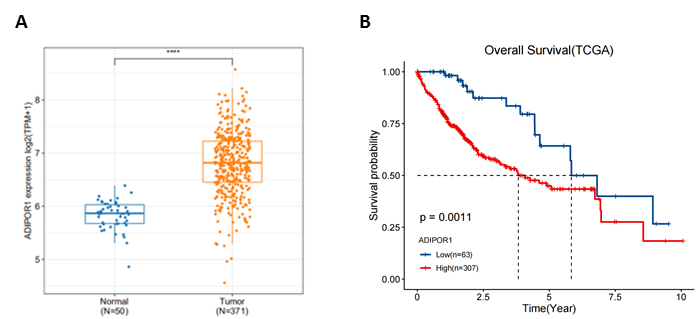


Fig S1. High expression levels of AdipoR1 was related to poor prognosis in HCC patients. (A) Comparison of AdipoR1 expression in different subgroup. A, non-Tumor and Tumor. (B) Overall survival of HCC patients with high and low AdipoR1 expression levels. (P=0.0033; LinkedOmics database, Kaplan-Meier survival analysis). Patients with high AdipoR1 levels had poor survival outcomes. Data is presented as the mean ± SD, *p < 0.05, ** p < 0.01, *** p < 0.001, **** p < 0.0001.

**
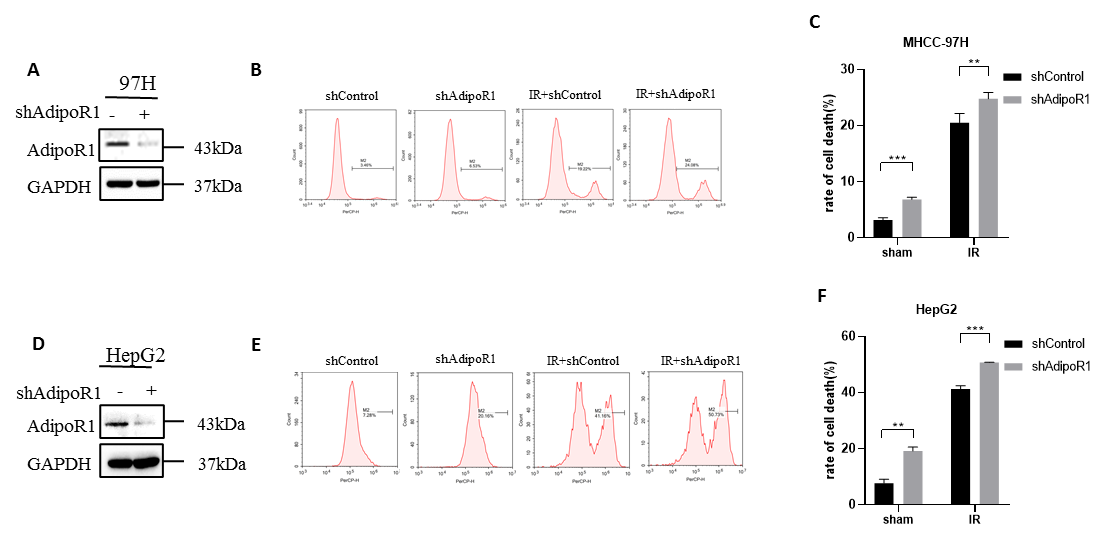
**

Fig S2. AdipoR1-knockdown increased IR-induced cell death in HCC cells. (A, D) AdipoR1 knock down was determined by Western blot in MHCC-97H (A) and HepG2 (D) cells. (B-F) After knock down of AdipoR1 by shRNA in MHCC-97H (B-C) and HepG2 (E-F) cells, followed by 10Gy radiation, cell death were assessed by flow cytometry using trypan blue. Data is presented as the mean ± SD, *p < 0.05, ** p < 0.01, *** p < 0.001.
